# Non-alcoholic fatty liver disease increases the risk of cardiovascular disease in young adults and children: a systematic review and meta-analysis of cohort studies

**DOI:** 10.3389/fcvm.2023.1291438

**Published:** 2024-01-10

**Authors:** Yan-Lin Liao, Gen-Yuan Zhu, Cheng Chang

**Affiliations:** ^1^ Department of Cardiovascular Medicine, Wuhan Hospital of Traditional Chinese Medicine, Wuhan, China; ^2^ Department of Gastroenterology, Wuhan Hospital of Traditional Chinese Medicine, Wuhan, China

**Keywords:** non-alcoholic fatty liver disease, cardiovascular disease, cohort studies, systematic review, meta-analysis, metabolic dysfunctionassociated fatty liver disease

## Abstract

**Background and aims:**

It is uncertain if there is a link between non-alcoholic fatty liver disease (NAFLD) and cardiovascular diseases (CVD) in young adults and children. To evaluate the potential link between these two conditions, we conducted a systematic review and meta-analysis of cohort studies.

**Methods:**

A comprehensive search was conducted in PubMed, Web of Science and Embase in order to locate all relevant cohort studies published until August 2023. Random effects meta-analyses were conducted using the generic inverse variance method, with additional subgroup and sensitivity analyses. The Newcastle-Ottawa Scale was employed to evaluate the methodological quality.

**Results:**

Four cohort studies (eleven datasets) involving 10,668,189 participants were included in this meta-analysis. This meta-analysis demonstrated that NAFLD increases the risk of CVD in young adults and children (HR = 1.63, 95% CI: 1.46–1.82, *P* < 0.00001). Further subgroup analyses showed that individuals with NAFLD were at a heightened risk of coronary heart disease (CHD) (HR = 3.10, 95% CI: 2.01–4.77, *P* < 0.00001), myocardial infarction (MI) (HR = 1.69, 95% CI: 1.61–1.78, *P* < 0.00001), atrial fibrillation (AF) (HR = 2.00, 95% CI: 1.12–3.57, *P* = 0.02), congestive heart failure (CHF) (HR = 3.89, 95% CI: 1.20–12.61, *P* = 0.02), and stroke (HR = 1.47, 95% CI: 1.39–1.55, *P* < 0.00001). The results of subgroup analyses based on the study location, NAFLD definition, and follow-up time also showed consistency with the overall results. Sensitivity analyses showed that our results were robust. All of the included studies were judged to be of medium to high quality.

**Conclusion:**

Current evidence reveals that NAFLD is linked to an increased risk of major CVD (including CHD, MI, AF, CHF and stroke) in young adults and children. Further research is needed to strengthen this association and provide stronger evidence for primary prevention of CVD in young adults and children with NAFLD.

**Systematic Review Registration:**

https://www.crd.york.ac.uk/PROSPERO/, PROSPERO registration number: CRD42023457817.

## 1 Introduction

Currently, non-alcoholic fatty liver disease (NAFLD) is the most common chronic liver disease worldwide, with a prevalence of up to 30 percent in adults and rising (1). NAFLD is also common in children and adolescents, accounting for 7.6 percent of all pediatric patients, with a prevalence of over 30 percent in overweight children. The prevalence of NAFLD has been observed to increase to 20 percent in young adults with a mean age of onset of 24 years, making it a major health problem worldwide (2–5). According to a recent national health and nutrition screening survey conducted in the United States between 2017 and 2018, a high prevalence of NAFLD and severe fibrosis was observed among adolescents, at 24.16% and 4.4%, respectively (6). The histological characteristics of NAFLD range from mild steatosis to non-alcoholic steatohepatitis (NASH), which, if not addressed, can cause fibrosis, cirrhosis, and hepatocellular carcinoma (7). Nevertheless, increasing evidence suggests that the effects of NAFLD are not limited to liver-related issues, but also extend to a range of other medical conditions, including cardiovascular diseases (CVD) (8, 9). CVD is a major determinant of mortality and poor prognosis in individuals with NAFLD, and is second only to liver cirrhosis as the potential cause of death, as well as the most common cause of death (10). CVD is considered to be a significant contributor to the outcome and progression of NAFLD, however, there is still considerable debate and disagreement about the potential links between NAFLD and CVD and how strong the relationship is.

Previous several meta-analyses (11–14) have been conducted to examine the association between NAFLD and cardiovascular events, and the results revealed that NAFLD was associated with a greater risk of major CVD (atrial fibrillation, heart failure, myocardial infarction, and stroke). Previous research has almost exclusively explored the relationship between NAFLD and CVD in middle-aged and senior citizens. However, the link between NAFLD and the possibility of CVD in young adults (age ≤ 40) and children is still uncertain.

In order to evaluate the relationship between NAFLD and the risk of CVD in young adults and children, we conducted a systematic review and meta-analysis of cohort studies. Considering the present global burden of NAFLD, determining the correlation between it and CVD in children and young NAFLD patients is of great clinical significance for their management.

## 2 Materials and methods

This research was pre-planned and registered on the PROSPERO platform (registration number. 42023457817). We complied with the PRISMA statement for Systematic Reviews and Meta-Analyses (15) while conducting this research.

### 2.1 Literature search strategy

A comprehensive search was conducted in PubMed, Web of Science and Embase in order to locate all relevant studies published until August 2023. Utilizing MeSH terms and free words for electronic computer retrieval. The detailed search strategies for three databases were outlined in Supplementary Table S1. To ensure comprehensiveness, all relevant preliminary research and review articles’ references are reviewed and supplementary research is sought.

### 2.2 Eligibility criteria

In order to be included in this study, the criteria were as follows: (1) all cohort studies investigating the risk of CVD among children and young adults with NAFLD compared to those without NAFLD, CVD including myocardial Infarction (MI), heart failure (HF), atrial fibrillation (AF), coronary heart disease (CHD), and stroke; (2) study participants were children and young adults (age ≤ 40 years old); (3) the outcome of interest is presented through Hazard Ratios (HRs) with 95% Confidence Intervals (CIs) in the reports. Excluded from the study were: (1) studies on adults aged over 40 years; (2) case-control or cross-sectional studies; (3) abstracts, editorials, comments, letters, reviews, or meta-analyses; (4) studies without relevant data. Two investigators independently reviewed all studies for eligibility. In cases of disagreement, they discussed and reached a resolution.

### 2.3 Data extraction and quality assessment

Data was independently collected and the methodological quality of each study was evaluated by two investigators (Liao YL and Zhu GY). In case of any discrepancies, consensus was reached. The first author's surname, publication year, country of origin, study subjects, participants' characteristics, confirmation methods of NAFLD and CVD, follow-up time, HRs with their 95% CI, and adjusted confounders were all extracted from each eligible study.

The Newcastle-Ottawa Scale (NOS) was employed to evaluate the quality of the cohort studies included in the assessment (16). This method utilizes a star rating system to rate a study in three areas: selection of participants (maximum of four stars), comparability of study groups (maximum of two stars), and ascertainment of outcomes of interest (maximum of three stars). This results in a maximum of nine stars, which is the highest quality. Studies with nine stars were considered to be of high-quality, those with seven or eight stars were judged to be of moderate-quality, and those with six or fewer stars were deemed to be of low-quality (12).

### 2.4 Statistical analysis

The Cochrane Collaboration's Review Manager software Version 5.3 (Copenhagen, Denmark) was utilized to conduct meta-analyses. The HRs and 95% CIs were used to measure the effect size of each eligible study. From studies that reported HRs with various levels of covariate adjustment, the HRs that demonstrated the most significant adjustment for potential confounders were identified. Utilizing the DerSimonian and Laird method (17), a random effects meta-analysis was conducted to estimate the pooled adjusted HRs of all eligible studies and generate an overall effect size. We employed the Cochran's *Q*-test (*P* < 0.10) and *I*^2^ statistic to assess statistical heterogeneity among studies. *I*^2^ values of 0%–25% suggest insignificant heterogeneity; those between 26%–50% suggest low heterogeneity; 51%–75% signify moderate heterogeneity; and 76%–100% signify high heterogeneity (18). Subgroup analyses were conducted to investigate if particular study and participant traits had an impact on the findings and detect potential sources of heterogeneity. To determine the stability of the results, a sensitivity analysis was performed by sequentially removing each of the included studies. To assess the risk of publication bias, Begg's funnel plot, Begg's (19) and Egger's tests (20) were executed using STATA/SE 12.0 (STATA Corporation, Texas, USA). All statistical tests were two-sided and any results with a *p*-value of less than 0.05 were deemed to be significant.

## 3 Results

### 3.1 Study selection

**Figure 1:**
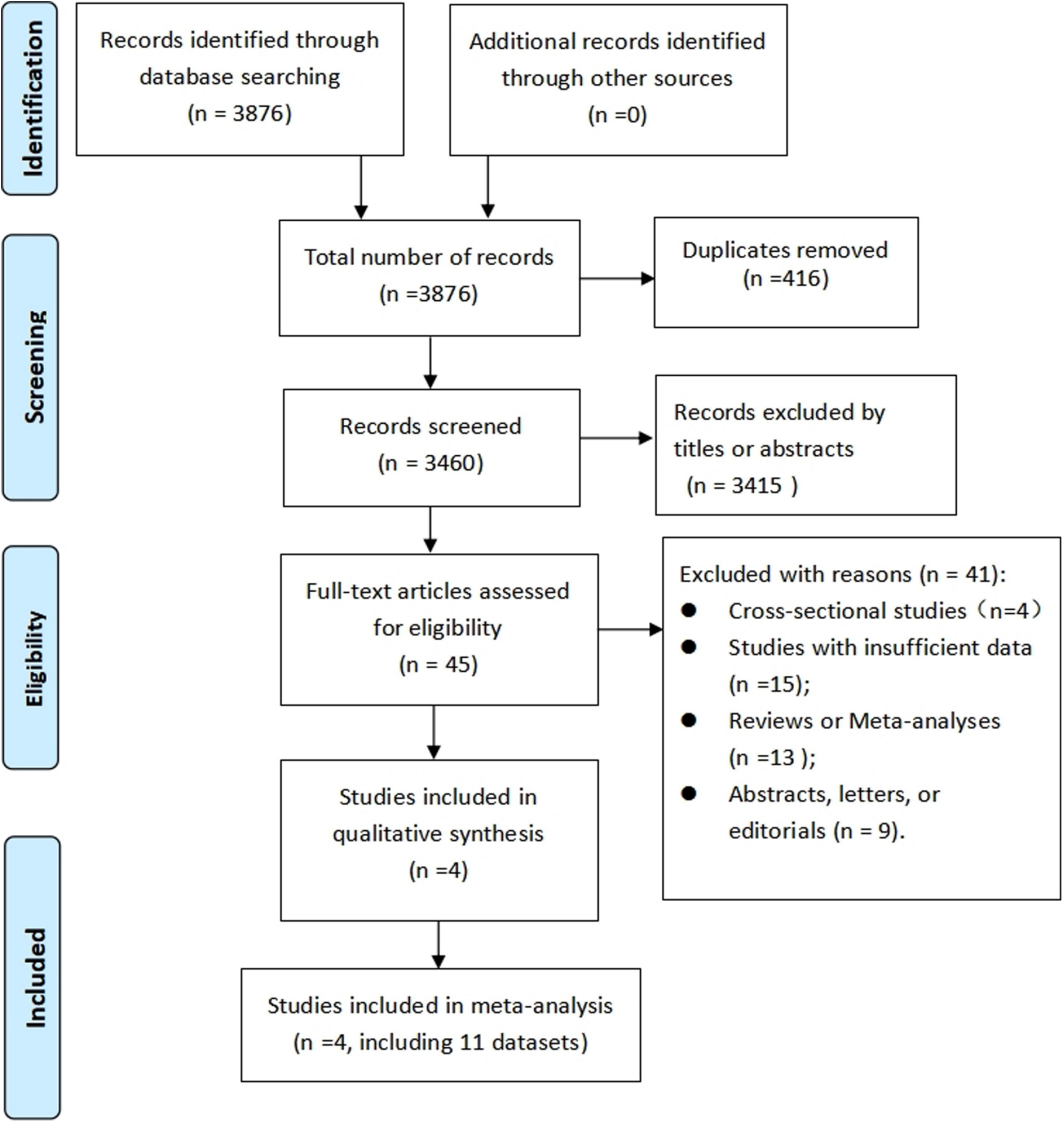
PRISMA flowchart of study selection process.

Following a computer system search, 3,876 records were revealed, with duplicates removed, resulting in 3,460 records. After further evaluation of the full text of 45 studies, four cohort studies (21–24) (eleven datasets) were found to fulfill the criteria (the reason for the exclusion of 41 articles was provided Supplementary Table S2) and thus included in this meta-analysis. As shown in Figure 1, the PRISMA flowchart illustrates the process of research selection.

### 3.2 Study characteristics

Table 1 presents a synopsis of the main characteristics of included studies. Our meta-analysis was composed of four cohort studies, which encompassed eleven datasets and 10,668,189 participants. The publication period ranges from 2020 to 2023. In South Korea, two studies (22, 23) were carried out, while in Germany (20) and Switzerland (24), one study each was conducted. Three studies (21–23) were conducted on young adults in the age range of 18–40, and one study (24) investigated children and young people aged ≤ 25 years (mean age: 17 years). Regarding definition of NAFLD, two studies (22, 23) used fatty liver index, one (24) used liver biopsy, and one (21) used international classification of diseases codes. Three studies reported data on AF and stroke, two on CHD and MI, and one on congestive heart failure (CHF). The average length of the follow-up period was between 7.4 and 16.6 years. The two studies (21, 24) that were given nine stars were rated as having high quality, while the other two (22, 23) were given eight stars, signifying moderate quality. A detailed analysis of the methodological quality of the studies included in the NOS is provided in the Supplementary Table S3 available on the web. Supplementary Table S4 contains the information about the study subjects, adjusted confounders and corresponding data.

**Table 1:** Main characteristics of included studies.

Study	Country	Sample size	Age range/average age (years)	Male (%)	NAFLD definition	Outcome reported	Follow-up time (mean years)	NOS score
Labenz, (21)	Germany	5 820	18–40	50.2	ICD codes	MI, CHD, AF, stroke	10	9
Choi, (22)	Korea	5 333 907	20–39	57.0	FLI (≥60)	AF	7.4	8
Chung, (23)	Korea	5 324 410	33	56.5	FLI (≥60)	MI, stroke	8.4	8
Simon, (24)	Sweden	4 052	17	62.8	Liver biopsy	CHD, CHF, AF, stroke	16.6	9

NAFLD, nonalcoholic fatty liver disease; FLI, fatty liver index; ICD, international classification of diseases; MI, myocardial infarction; CHD, coronary heart disease; AF, atrial fibrillation; CHF, congestive heart failure; NOS, newcastle-ottawa scale.

### 3.3 Meta analysis of the relationship between NAFLD and CVD

**Figure 2:**
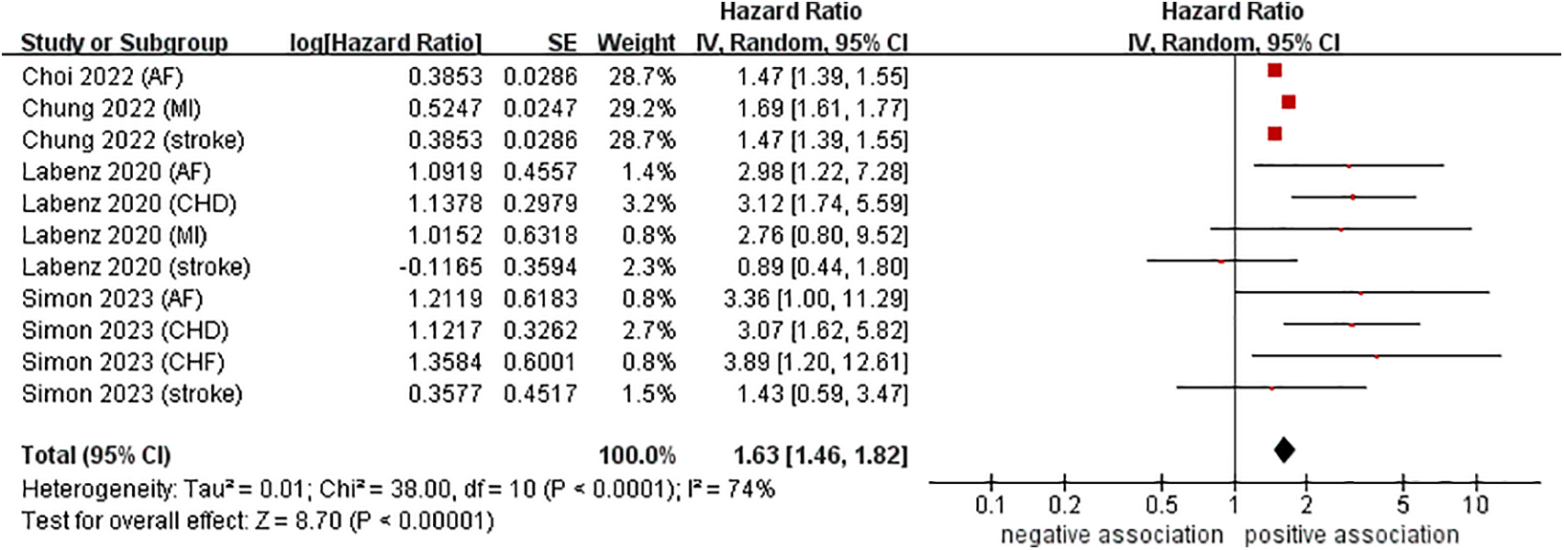
Forest plot of association between NAFLD and risk of incident CVD in young adults and children.

Data from four cohort studies (eleven datasets) (21–24) showed a link between NAFLD and the risk of developing CVD. This meta-analysis, based on random-effects model, revealed that NAFLD increases the risk of CVD in young adults and children (pooled HR = 1.63, 95% CI: 1.46–1.82, *P* < 0.00001). We observed a moderate heterogeneity (*I*^2^ = 74%, *P* < 0.0001) (Figure 2).

### 3.4 Subgroup analyses and sensitivity analyses

**Figure 3:**
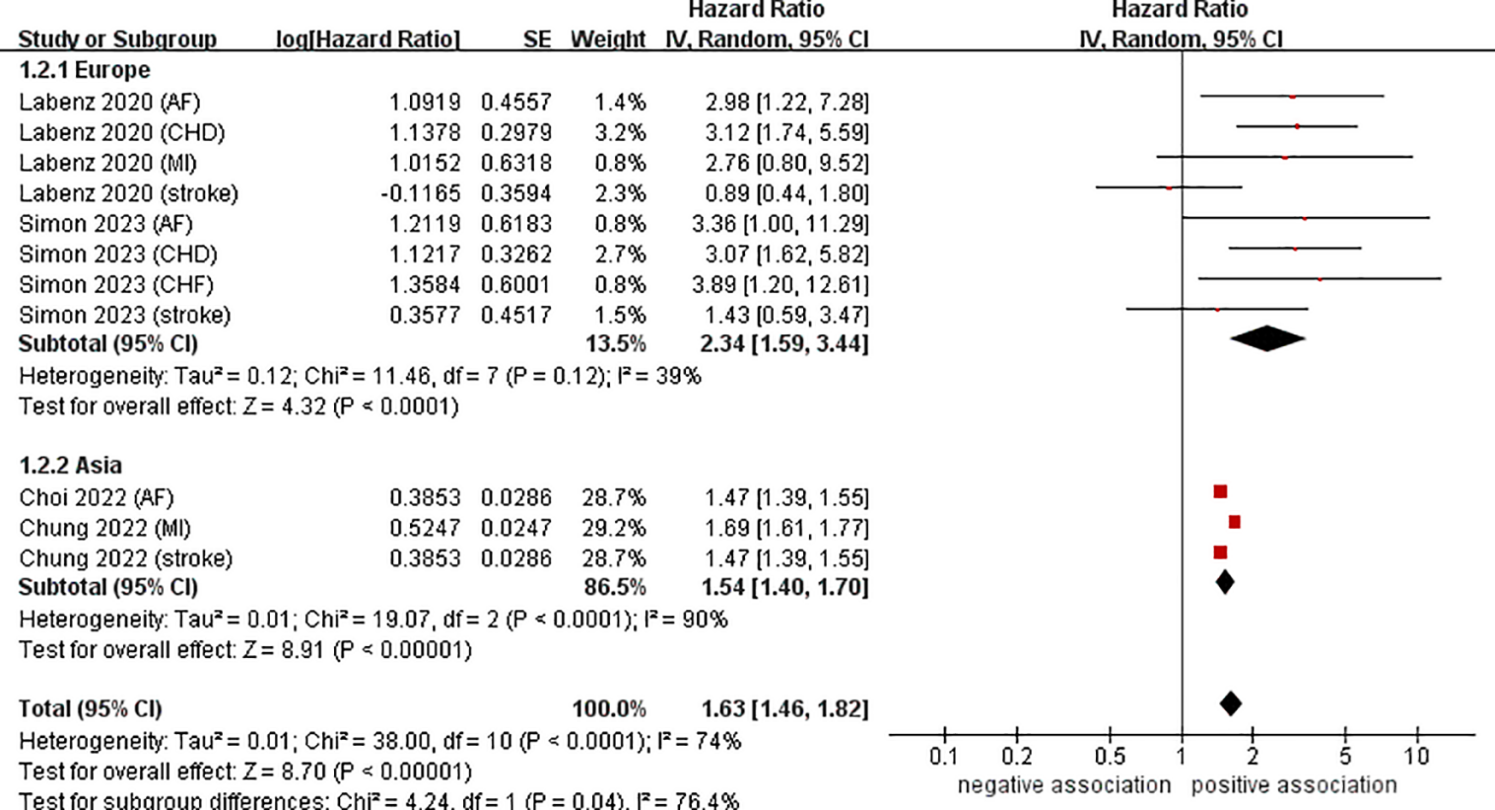
Forest plot of subgroup analysis based on study location.

**Figure 4:**
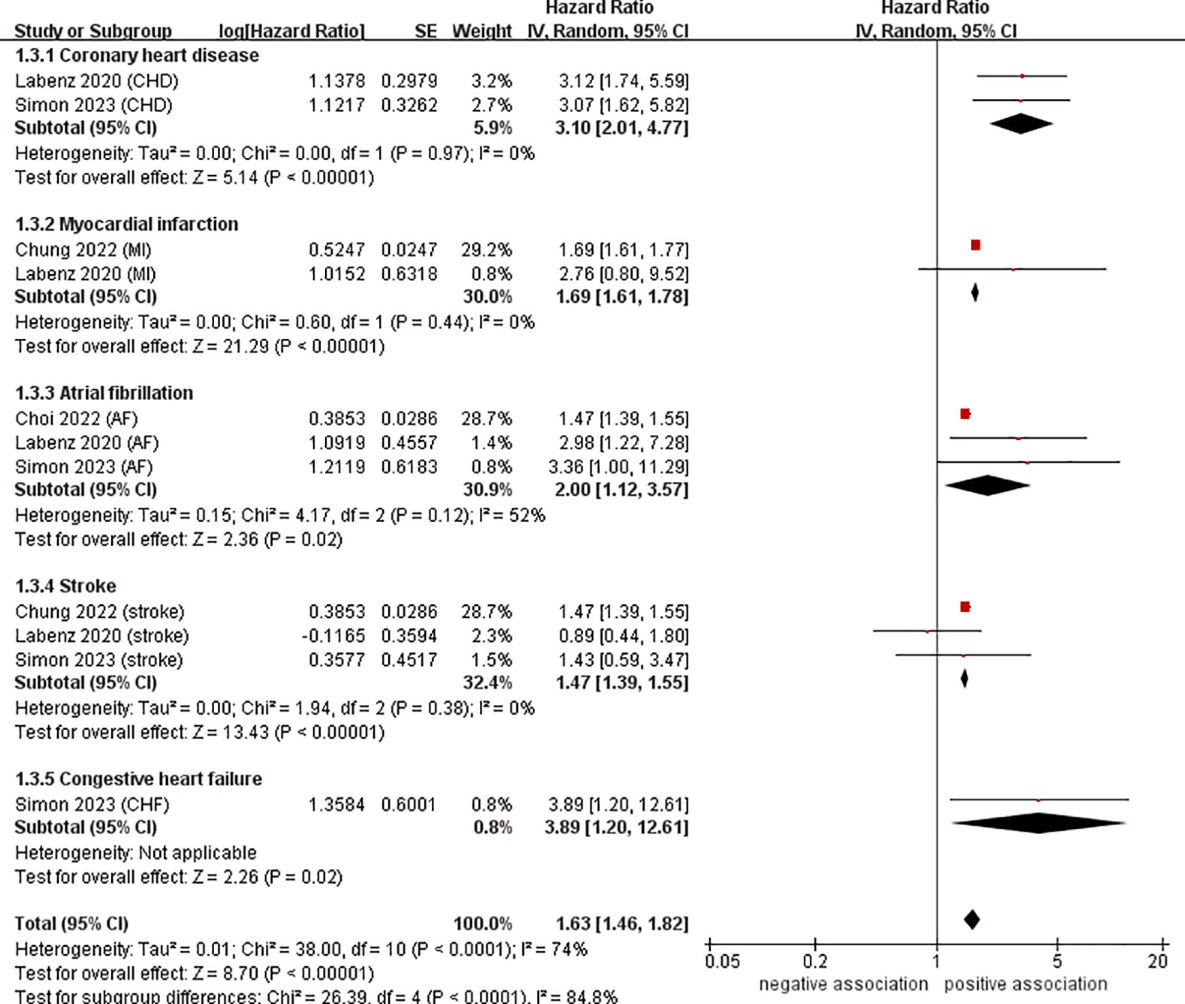
Forest plot of subgroup analysis based on types of CVD.

**Figure 5:**
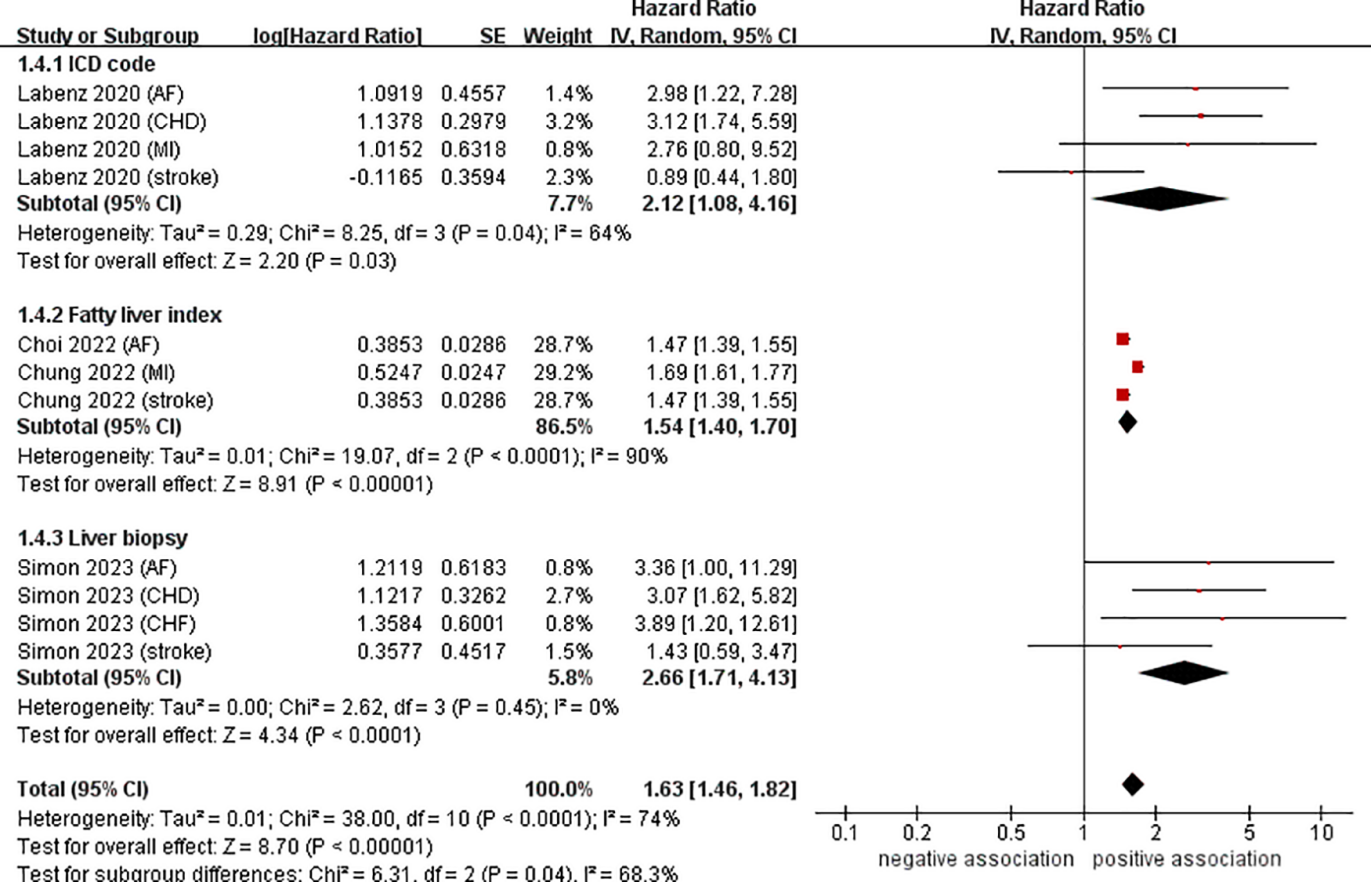
Forest plot of subgroup analysis based on definition of NAFLD.

**Figure 6:**
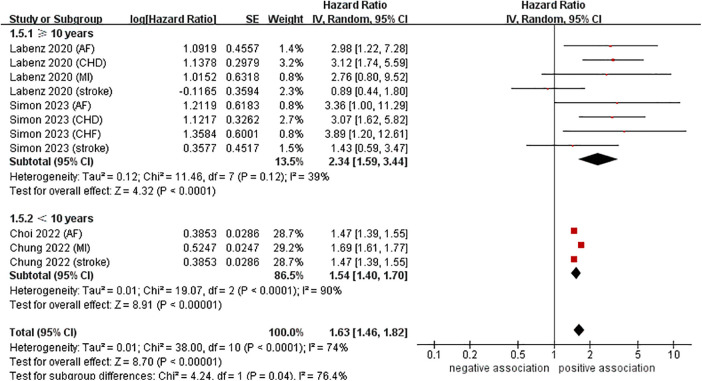
Forest plot of subgroup analysis based on follow-up time.

To investigate the sources of heterogeneity among the studies included and any possible factors that may impact the overall results, we conducted numerous subgroup analyses based on study location, types of CVD, definition of NAFLD, and follow-up time (Table 2). Results from the subgroup analysis based on the study location showed that NAFLD was linked to a heightened risk of CVD both in Europe (*n* = 2, HR = 2.34, 95% CI: 1.59–3.44, *P* < 0.0001; *I*^2^ = 39%) and in Asia (*n* = 2, HR = 1.54, 95% CI: 1.40–1.70, *P* < 0.0001; *I*^2^ = 90%) (Figure 3). Through a subgroup analysis of different types of CVD, it was observed that those with NAFLD were at a heightened risk of CHD (*n* = 2, HR = 3.10, 95% CI: 2.01–4.77, *P* < 0.00001; *I*^2^ = 0%), MI (*n* = 2, HR = 1.69, 95% CI: 1.61–1.78, *P* < 0.00001; *I*^2^ = 0%), AF (*n* = 3, HR = 2.00, 95% CI: 1.12–3.57, *P* = 0.02; *I*^2^ = 52%), CHF (*n* = 1, HR = 3.89, 95% CI:1.20–12.61, *P* = 0.02), and stroke (*n* = 3, HR = 1.47, 95% CI: 1.39–1.55, *P* < 0.00001; *I*^2^ = 0%) (Figure 4). It was revealed through the results that the heterogeneity of the different subgroups had decreased to various levels, signifying that types of CVD could be a source of statistical heterogeneity. The subgroup analyses that employed the definition of NAFLD and follow-up time yielded results that were in agreement with the overall results (Figures 5, 6).

**Table 2:** The results of subgroup analyses.

Subgroups	No. of studies	HR (95% CI)	*P*_association_	*I^2^*(%)	*P*_heterogeneity_
Study location					
Europe	2	2.34 (1.59–3.44)	<0.0001	39	0.12
Asia	2	1.54 (1.40–1.70)	<0.00001	90	<0.0001
Types of CVD					
Coronary heart disease	2	3.10 (2.01–4.77)	<0.00001	0	0.97
Myocardial infarction	2	1.69 (1.61–1.78)	<0.00001	0	0.44
Atrial fibrillation	3	2.00 (1.12–3.57)	0.02	52	0.12
Stroke	3	1.47 (1.39–1.55)	<0.00001	0	0.38
Congestive heart failure	1	3.89 (1.20–12.61)	0.02	-	-
Definition of NAFLD					
ICD code	1	2.12 (1.08–4.16)	0.03	64	0.04
FLI	2	1.54 (1.40–1.70)	<0.00001	90	<0.0001
Liver biopsy	1	2.66 (1.71–4.13)	<0.0001	0	0.45
Follow-up time					
≥10 years	2	2.34 (1.59–3.44)	<0.0001	39	0.12
<10 years	2	1.54 (1.40–1.70)	<0.00001	90	<0.0001
Overall studies	4 (11 datasets)	1.63 (1.46–1.82)	<0.00001	74	<0.0001

CVD, cardiovascular disease; HR, hazard ratio; CI, confidence interval; ICD, international classification of diseases; FLI, fatty liver index.

To evaluate the reliability of our results, we conducted sensitivity analysis by leaving out one study at a time. As Table 3 demonstrates, our research results remain unchanged when any particular studies were excluded, suggesting that our results were robust (Supplementary Figure S1).

**Table 3:** Results of sensitivity analyses.

Studies omitted	HR (95% CI)	*P*_association_	Heterogeneity
Labenz (21) (AF)	1.61 (1.45–1.80)	<0.00001	*I^2 ^*= 75%*, P *< 0.0001
Labenz (21) (CHD)	1.59 (1.43–1.76)	<0.00001	*I^2 ^*= 72%*, P *= 0.0002
Labenz (21) (MI)	1.62 (1.45–1.81)	<0.00001	*I^2 ^*= 76%*, P *< 0.0001
Labenz (21) (stroke)	1.65 (1.48–1.84)	<0.00001	*I^2 ^*= 75%*, P *< 0.0001
Choi (22) (AF)	1.74 (1.49–2.02)	<0.00001	*I^2 ^*= 72%*, P *= 0.0002
Chung (23) (MI)	1.60 (1.41–1.81)	<0.00001	*I^2 ^*= 57%*, P *= 0.01
Chung (23) (stroke)	1.74 (1.49–2.02)	<0.00001	*I^2 ^*= 72%*, P *= 0.0002
Simon (24) (AF)	1.62 (1.45–1.81)	<0.00001	*I^2 ^*= 75%*, P *< 0.0001
Simon (24) (CHD)	1.60 (1.43–1.77)	<0.00001	*I^2 ^*= 73%*, P *= 0.0001
Simon (24) (CHF)	1.61 (1.45–1.80)	<0.00001	*I^2 ^*= 75%*, P *< 0.0001
Simon (24) (stroke)	1.63 (1.46–1.83)	<0.00001	*I^2 ^*= 76%*, P *< 0.0001

MI, myocardial infarction; CHD, coronary heart disease; AF, atrial fibrillation; CHF, congestive heart failure; HR, hazard ratio; CI, confidence interval.

### 3.5 Publication bias assessment

A slight asymmetry in the Begg's funnel plot for the overall results was observed (Supplementary Figure S2). However, no significant publication bias was revealed by the Begg's test and Egger's test (*P*_Begg _= 0.938, *P*_Egger _= 0.210).

## 4 Discussion

### 4.1 Principle findings of this meta-analysis

As far as we know, this meta-analysis is the first to investigate the correlation between NAFLD and the risk of CVD in both young adults and children. Our meta-analysis, comprising of four cohort studies and 11 datasets, included 10,668,189 participants and a median follow-up of 10.6 years. We found that NAFLD significantly increases the risk of CVD in both young adults and children, with a pooled HR of 1.63 (95% CI: 1.46–1.82). Further analyses conducted in different locations, on different types of CVD, using different definitions of NAFLD, and over different follow-up times, revealed that the risk associated remained largely unchanged. Additional sensitivity analyses corroborated the stability of our conclusions.

### 4.2 Potential explanations and clinical implication

The exact mechanisms connecting NAFLD to CVD remain unclear. At present, there are a few potential explanations. Firstly, systemic inflammation may be a key factor in connecting NAFLD and CVD at a pathophysiological level. NAFLD is characterized by low-grade systemic inflammation (9). It has been observed that long-term, low-grade inflammation of the liver can cause diseases outside of the organ (25). Young adults who are obese are particularly vulnerable to developing NAFLD, which is believed to be the initial phase of a long-term metabolic and inflammatory disorder. Leukocytes in adipose tissue create a prolonged proinflammatory process that impairs adipocyte insulin sensitivity and contributes to insulin resistance, which can cause harm to the myocardium (26, 27). Studies are increasingly demonstrating that proinflammatory mediators (including tumor necrosis factor, IL-6, and C-reactive protein) may be the cause of changes in the structure and functioning of the heart (28). Secondly, oxidative stress triggered by NAFLD may lead to an increased potential for arrhythmias. Systemic oxidative stress may be caused by NAFLD, which can lead to an increase in reactive oxygen species in the liver and a change in homocysteine metabolism, thereby raising the chance of atrial and ventricular arrhythmias (28). Thirdly, NAFLD, particularly NASH with an ever-growing amount of liver fibrosis, can have a detrimental effect on insulin resistance, promote atherogenic dyslipidaemia, and trigger the emission of pro-inflammatory cytokines and pro-atherogenic mediators, thus increasing the likelihood of CVD (9, 29, 30). Fourthly, Liver dysfunction caused by NAFLD can cause thrombotic vascular disease, as it changes the production of coagulation proteins, lipoproteins, and factors related to inflammation (31). Fifthly, it has been suggested that NAFLD could be associated with modifications in the structure of the left ventricle and an enlargement of the left atrium, which may be contributory to the emergence of AF and HF (11). Finally, recent research has revealed that gut microbes and their associated metabolites may be significant contributors to CVD. Lipopolysaccharide and other substances produced by intestinal dysbiosis in the gut may be the cause of atherosclerosis, changes in the structure of the heart muscle and valves, and even abnormalities in the heart's electrical conduction system (9, 32).

Research has demonstrated a definite connection between NAFLD and the potential for CVD in both young adults and children. Considering the present global burden of NAFLD, we hold the view that the results of our meta-analysis have considerable significance in clinical practice. These results suggest that further research should be conducted to enhance CVD screening and risk stratification techniques for young adults and children with NAFLD who may benefit from early CVD prevention.

### 4.3 Comparison with previous studies

Several previous larger meta-analyses (11–14, 33) have demonstrated a link between NAFLD and risk of CVD. Alon et al. (11) included 20 studies (17 cohort and 3 case-control studies) and showed that NAFLD were more likely to experience MI, AF, HF, and ischemic stroke; however, the intensity of this association may be moderated by age, sex, and the characteristics of the study. In a meta-analysis conducted by Mantovani et al. (12) involving 36 cohort studies with 5,802,226 participants, aged 53 on average, it was revealed that NAFLD was connected with a moderately increased risk of fatal or non-fatal CVD events (HR = 1.45, 95% CI = 1.31–1.61). As the progression of NAFLD became more pronounced, particularly in the case of fibrosis, the risk of CVD events rose significantly (HR = 2.50, 95% CI = 1.68–3.72), irrespective of age, sex, diabetes, adiposity measures, and other usually observed cardiometabolic risk factors. Three additional meta-analyses (13, 14, 33) investigated the correlation NAFLD and specific CVD. Wang et al. (13) performed a meta-analysis of 20 studies (15 cohort, 1 case-control, and 4 cross-sectional studies) and concluded that NAFLD had a slightly increased risk of stroke (*n* = 18, OR = 1.18, 95% CI: 1.08–1.30, *P* = 0.0005). Nevertheless, the evidence was not strong enough to confirm the relationship between NAFLD-fibrosis and stroke (*n* = 3, OR = 1.37, 95% CI: 0.99–1.91, *P* = 0.06). In 2022, Mantovani and colleagues (33), who included 11 longitudinal cohort studies involving 11 242 231 middle-aged individuals, demonstrated that NAFLD was linked to a moderately increased risk of developing HF for the first time (HR = 1.50, 95% CI: 1.34–1.67, *P* < 0.0001), and this risk was not affected by factors such as age, sex, obesity, ethnicity, hypertension, diabetes, or other common cardiovascular risk factors. Recently, Zhou et al. (14) carried out a meta-analysis comprising 12 cohort studies, which involved 14 213 289 participants. The results of the study demonstrated a statistically significant correlation between NAFLD and the development of AF (HR = 1.18, 95% CI: 1.12–1.23, *P* < 0.00001).

In comparison to all the previous meta-analyses, this study further confirms and expands upon the previous findings. For one thing, previous studies mainly concentrated on middle-aged and elderly individuals, who have a high risk of CVD, whereas our study focused on young adults and children, providing the most recent data on the prevention of CVD in those with NAFLD. For another, we only included cohort studies, avoiding cross-sectional and case-control study designs that can be more vulnerable to bias.

### 4.4 Strengths and limitations

Our study demonstrates several noteworthy strengths. First, as noted previously, this is the first meta-analysis to investigate the link between NAFLD and the risk of CVD in young adults and children, thus providing up-to-date evidence for primary preventative measures for CVD in those with NAFLD. Second, we implemented a few subgroup and sensitivity analyses to reinforce the association, thereby increasing the credibility of our results. Third, our meta-analysis includes studies of a medium to high quality, and the publication bias test showed no substantial publication bias, strengthening the reliability of the findings.

Despite this, this study has certain limitations. Firstly, because the studies were observational, it is not possible to conclude a causal relationship. Secondly, almost all studies adjusted for age, sex, obesity measures, and other cardiometabolic risk factors; however, these adjustments were not always consistent or complete, and unmeasured or residual confounders might still be present. Thirdly, despite the fact that the studies included in our meta-analysis were of medium to high quality, our meta-analysis revealed moderate heterogeneity (*I*^2^ = 74%), which could potentially reduce the reliability of our research results. To address this, we employed a random effects model which accounted for the discrepancies between studies. Furthermore, we performed multiple subgroup and sensitivity analyses to investigate the potential sources of the statistical heterogeneity, and found that different types of CVD may be a source of statistical heterogeneity. Fourthly, we had restricted data on the severity and development of NAFLD (NASH, liver fibrosis or cirrhosis), along with details on treatments and possible other factors, such as socio-demographical variables. We deem that these factors could have an effect on the connection between NAFLD and CVD, and more research is essential to comprehend their effect on the course of NAFLD patients. Fifthly, the criteria for diagnosing NAFLD are not uniform, which could have influenced the results. Sixthly, it is well-known that insulin resistance can cause an increase in the trygliceride pool in the liver, thus promoting the development of NAFLD. Novel lipid-lowering therapies (LLT) has been proposed as a potential treatment for those with hypercholesterolemia and NAFLD (34). The cohort studies we included did not report information on whether LLT was used in the participants, which may affect the relationship between NAFLD and CVD. Seventhly, recently, it has been observed that new terms such as metabolic dysfunction-associated fatty liver disease (MAFLD) and metabolic dysfunction-associated steatotic liver disease (MASLD) have been introduced. Studies have revealed that the prevalence of disease is not significantly affected by changes in diagnostic criteria (35, 36), yet patients diagnosed with MAFLD alone have a higher cardiovascular mortality rate than those diagnosed with NAFLD alone (37). It is yet to be determined if this alteration in diagnostic criteria is linked to cardiovascular events in children and young adults. Therefore, future studies should be conducted to explore the link between MAFLD/MASLD and CVD events based on the new diagnostic criteria. Finally, despite our large sample size of 10,668,189 participants, the majority of the population was from Asia and Europe, leaving out research from other regions. Consequently, the results may not be applicable to other populations. With the limited number of studies included, it is important to validate the above findings through future studies that involve different populations from various regions.

## 5 Conclusions

Current evidence reveals that NAFLD is linked to an increased risk of major CVD (including CHD, MI, AF, CHF and stroke) in young adults and children. Further research is needed to strengthen this association and provide stronger evidence for primary prevention of CVD in young adults and children with NAFLD.

## Data Availability

The original contributions presented in the study are included in the article/Supplementary Material, further inquiries can be directed to the corresponding author.
