# Supplementary material for: Non-alcoholic fatty liver disease increases the risk of cardiovascular disease in young adults and children: a systematic review and meta-analysis of cohort studies
Source: Front Cardiovasc Med. 2024 Jan 10;10:1291438. doi: 10.3389/fcvm.2023.1291438 (PMC10806083; doi:10.3389/fcvm.2023.1291438)

Supplementary online material

Table S1. The details of search strategy of three electronic databases

Table S2. Description of excluded studies

Table S3. Methodological quality assessment of included studies with NOS

Table S4. The other characteristics of included studies

Figure S1. The figure of sensitivity analysis (leave-one-out analysis)

Figure S2. The Begg's funnel plot for overall results

Table S1 The details of search strategy of three electronic databases (from database inception to August 24, 2023)	
**Databases**	**Search strings**
**PubMed**	("non-alcoholic fatty liver disease"[MeSH Terms] OR "non-alcoholic fatty liver disease"[All Fields] OR "nonalcoholic fatty liver disease"[All Fields] OR "non-alcoholic fatty liver"[All Fields] OR "nonalcoholic fatty liver"[All Fields] OR "nonalcoholic steatohepatitis"[All Fields] OR "non-alcoholic steatohepatitis"[All Fields] "fatty liver"[MeSH Terms] OR "fatty liver"[All Fields] OR "Metabolic dysfunction-associated fatty liver disease"[All Fields] OR "Metabolic associated fatty liver disease"[All Fields] OR "Metabolic Dysfunction-Associated Steatotic Liver Disease"[All Fields] OR NAFLD[All Fields] OR NASH[All Fields] OR NAFL[All Fields] OR MAFLD[All Fields] OR MASLD[All Fields]) AND ("myocardial infarction"[MeSH Terms] OR "myocardial infarction"[All Fields] OR "coronary heart disease"[All Fields] OR "ischaemic heart disease"[All Fields] OR "heart failure"[MeSH Terms] OR "heart failure"[All Fields] OR "congestive heart failure"[All Fields] OR "atrial fibrillation"[MeSH Terms] OR "atrial fibrillation"[All Fields] OR "cardiovascular diseases"[MeSH Terms] OR "cardiovascular disease"[All Fields] OR "stroke"[MeSH Terms] OR stroke[All Fields] OR "cerebrovascular disease"[All Fields] OR "cerebrovascular accident"[All Fields]) AND (children[All Fields] OR youngster[All Fields] OR "young adult"[All Fields])
**EMBASE**	#1 'non-alcoholic fatty liver disease'/exp OR 'non-alcoholic fatty liver disease'<br>#2 'nonalcoholic fatty liver disease '/exp OR 'nonalcoholic fatty liver disease'<br>#3 'nonalcoholic fatty liver'/exp OR 'nonalcoholic fatty liver'<br>#4 'non-alcoholic fatty liver'/exp OR 'non-alcoholic fatty liver'<br>#5 'nonalcoholic steatohepatitis'/exp OR 'nonalcoholic steatohepatitis'<br>#6 'non-alcoholic steatohepatitis'/exp OR 'non-alcoholic steatohepatitis'<br>#7 'fatty liver'/exp OR 'fatty liver'<br>#8 'metabolic dysfunction-associated fatty liver disease' OR 'metabolic dysfunction-associated steatotic liver disease'<br>#9 'metabolic associated fatty liver disease'<br>#10 'NAFLD' OR 'NASH' OR 'NAFL' OR 'MAFLD' OR 'MASLD'<br>#11 #1 OR #2 OR #3 OR #4 OR #5 OR #6 OR #7 OR #8 OR #9 OR #10<br>#12 'myocardial infarction'/exp OR 'myocardial infarction'

	#13 'coronary heart disease'/exp OR 'coronary heart disease'<br>#14 'ischaemic heart disease'/exp OR 'ischaemic heart disease'<br>#15 'heart failure'/exp OR 'heart failure'<br>#16 'congestive heart failure'/exp OR 'congestive heart failure'<br>#17 'atrial fibrillation'/exp OR 'atrial fibrillation'<br>#18 'cardiovascular disease'/exp OR 'cardiovascular disease'<br>#19 'stroke'/exp OR stroke<br>#20 'cerebrovascular disease'/exp OR 'cerebrovascular disease'<br>#21 'cerebrovascular accident'/exp OR 'cerebrovascular accident'<br>#22 #12 OR #13 OR #14 OR #15 OR #16 OR #17 OR #18 OR #19 OR #20 OR #21<br>#23 children OR youngster OR "young adult"<br>#24 #11 AND #22 AND #23
**Web of Science**	TS=("non-alcoholic fatty liver disease" OR "nonalcoholic fatty liver disease" OR "non-alcoholic fatty liver" OR "nonalcoholic fatty liver" OR "non-alcoholic steatohepatitis" OR "nonalcoholic steatohepatitis" OR "fatty liver" OR "Metabolic dysfunction-associated fatty liver disease" OR "Metabolic associated fatty liver disease" OR "Metabolic dysfunction-associated steatotic liver disease" OR NAFLD OR NASH OR NAFL OR MAFLD OR MASLD) AND TS=("myocardial infarction" OR "coronary heart disease" OR "ischaemic heart disease" OR "heart failure" OR "congestive heart failure" OR "atrial fibrillation" OR "cardiovascular disease" OR stroke OR "cerebrovascular disease" OR "cerebrovascular accident") AND TS=(children OR youngster OR "young adult")

Table S2. Description of excluded studies at the stage of eligibility according to the PRISMA flow chart			
**No.**	**First author**	**Publication year**	**Reason for exclusion**
1.	Ong	2008	studies with insufficient data
2.	Schwimmer	2008	studies with insufficient data
3.	Rubinstein	2008	review
4.	Misra	2009	review
5.	Liu	2010	studies with insufficient data
6.	García Roldán	2011	studies with insufficient data
7.	Pacifico	2011	review
8.	Ying	2011	review
9.	Domanski	2012	studies with insufficient data
10.	Gökçe	2013	studies with insufficient data
11.	Sert	2013	studies with insufficient data
12.	Sanches	2014	studies with insufficient data
13.	Van	2014	conference Abstract
14.	Baskar	2015	review
15.	Bonci	2015	meta-analysis
16.	Dogan	2015	cross-sectional study
17.	Koot	2015	cross-sectional study
18.	Rutigliano	2015	conference Abstract
19.	Kaczorowska	2016	studies with insufficient data
20.	Goisbault	2016	conference Abstract
21.	Mantovani	2016	review
22.	Mikolasevic	2016	cross-sectional study
23.	Bajaj	2017	conference Abstract
24.	Di Sessa	2017	review
25.	Ahmad	2018	cross-sectional study

26.	González-Ruíz	2018	studies with insufficient data
27.	Vanwagner	2018	conference Abstract
28.	Golabi	2019-	conference Abstract
29.	Liu	2019	meta-analysis
30.	Moreno-Del	2019	review
31.	Chociej	2020	review
32.	Erdol	2020	letter
33.	Tawadros	2020	conference Abstract
34.	Pennisi	2021	studies with insufficient data
35.	Xu	2021	studies with insufficient data
36.	Forlano	2021	review
37.	Han	2021	studies with insufficient data
38.	Koulaouzidis	2021	meta-analysis
39.	Maniak	2021	conference Abstract
40.	Jin	2022	studies with insufficient data
41.	Ishido	2023	studies with insufficient data

References for the table

1. Ong JP, Pitts A, Younossi ZM. Increased overall mortality and liver-related mortality in non-alcoholic fatty liver disease. J Hepatol 2008;49:608-612.

2. Schwimmer JB, Pardee PE, Lavine JE, Blumkin AK, Cook S. Cardiovascular risk factors and the metabolic syndrome in pediatric nonalcoholic fatty liver disease. Circulation. 2008 Jul 15;118(3):277-83.

3. Rubinstein E, Lavine JE, Schwimmer JB. Hepatic, cardiovascular, and endocrine outcomes of the histological subphenotypes of nonalcoholic fatty liver disease. Semin Liver Dis. 2008 Nov;28(4):380-5.

4. Misra VL, Khashab M, Chalasani N. Nonalcoholic fatty liver disease and cardiovascular risk. Curr Gastroenterol Rep 2009;11:50-55.

5. Liu LR, Fu JF, Liang L, Huang K. Relationship between nonalcoholic fatty liver disease and cardiovascular disease in children with obesity. Chinese Journal of Contemporary Pediatrics 2010;12:547-550.

6. García Roldán R, Marén Altabás Y. Cardiovascular risk factors associated with the metabolic syndrome in obese adolescents. Journal of Diabetes 2011;3:146.

7. Pacifico L, Nobili V, Anania C, Verdecchia P, Chiesa C. Pediatric nonalcoholic fatty liver disease, metabolic

syndrome and cardiovascular risk. World J Gastroenterol. 2011 Jul 14;17(26):3082-91.

8. Ying I, Saposnik G, Vermeulen MJ, Leung A, Ray JG. Nonalcoholic fatty liver disease and acute ischemic stroke. Epidemiology. 2011 Jan;22(1):129-30.

9. Domanski JP, Park SJ, Harrison SA. Cardiovascular disease and nonalcoholic fatty liver disease: does histologic severity matter? J Clin Gastroenterol. 2012 May-Jun;46(5):427-30.

10. Gökçe S, Atbinici Z, Aycan Z, Cınar HG, Zorlu P. The relationship between pediatric nonalcoholic fatty liver disease and cardiovascular risk factors and increased risk of atherosclerosis in obese children. Pediatr Cardiol. 2013 Feb;34(2):308-15.

11. Sert A, Pirgon O, Aypar E, Yilmaz H, Odabas D. Subclinical hypothyroidism as a risk factor for the development of cardiovascular disease in obese adolescents with nonalcoholic fatty liver disease. Pediatr Cardiol. 2013 Jun;34(5):1166-74.

12. Sanches PL, de Piano A, Campos RM, Carnier J, de Mello MT, Elias N, Fonseca FA, Masquio DC, da Silva PL, Corgosinho FC, Tock L, Oyama LM, Tufik S, Dâmaso AR. Association of nonalcoholic fatty liver disease with cardiovascular risk factors in obese adolescents: the role of interdisciplinary therapy. J Clin Lipidol. 2014 May-Jun;8(3):265-72.

13. VanWagner LB, Wilcox JE, Colangelo LA, Lloyd-Jones DM, Carr JJ, Lima JA, Lewis CE, Rinella ME, Shah SJ. Association of nonalcoholic fatty liver disease with subclinical myocardial remodeling and dysfunction: A population-based study. Hepatology. 2015 Sep;62(3):773-83.

14. Baskar S, Jhaveri S, Alkhouri N. Cardiovascular risk in pediatric nonalcoholic fatty liver disease: recent advances. Clinical Lipidology 2015;10:351-362.

15. Bonci E, Chiesa C, Versacci P, Anania C, Silvestri L, Pacifico L. Association of Nonalcoholic Fatty Liver Disease with Subclinical Cardiovascular Changes: A Systematic Review and Meta-Analysis. Biomed Res Int. 2015;2015:213737.

16. Dogan S, Celikbilek M, Yilmaz YK, Sarikaya S, Zararsiz G, Serin HI, Borekci E, Akyol L, Pirti I, Davarci SE. Association between liver fibrosis and coronary heart disease risk in patients with nonalcoholic fatty liver disease. Eur J Gastroenterol Hepatol. 2015 Mar;27(3):298-304.

17. Koot BG, de Groot E, van der Baan-Slootweg OH, Bohte AE, Nederveen AJ, Jansen PL, Stoker J, Benninga MA. Nonalcoholic fatty liver disease and cardiovascular risk in children with obesity. Obesity (Silver Spring). 2015 Jun;23(6):1239-43.

18. Rutigliano I, De Filippo G, Vinci R, Kyheng C, Petraccaro A, Conoscitore M, Pettoello-Mantovani M, et al. Hepatic steatosis influences significantly the cardiovascular risk in children with metabolic syndrome. Hormone Research in Paediatrics 2015;84:416.

19. Kaczorowska M, Ryterska K, Ossowski P, Maciejewska D, Sabinicz A, Jamioł-Milc D, Raszeja -Wyszomirska J, et al. Metabolic risk factors of coronary heart disease in relation to anthropometric measures in nonalcoholic fatty liver disease patients following dietary intervention. Pomeranian journal of life sciences 2016;62:8-14.

20. Goisbault M, Legrand A, Storey C, Bonnet D, Tounian P, Dubern B. Liver disease and early vascular lesions in obese children. Journal of Pediatric Gastroenterology and Nutrition 2016;62:791.

21. Mantovani A, Ballestri S, Lonardo A, Targher G. Cardiovascular Disease and Myocardial Abnormalities in Nonalcoholic Fatty Liver Disease. Dig Dis Sci. 2016 May;61(5):1246-67.

22. Mikolasevic I, Milic S, Racki S, Zaputovic L, Stimac D, Radic M, Markic D, Orlic L. Nonalcoholic Fatty Liver Disease (NAFLD)-A New Cardiovascular Risk Factor in Peritoneal Dialysis Patients. Perit Dial Int. 2016 Jul-Aug;36(4):427-32.

23. Bajaj S, Prajapati SK, Saxena P, Yadav RK, Misra V, Nath B. Association of nonalcoholic fatty liver disease and metabolic syndrome with cardiovascular risk factors and atherosclerosis. Indian Journal of Endocrinology and Metabolism 2017;21:S38-S39.

24. Di Sessa A, Umano GR, Miraglia Del Giudice E. The Association between Non-Alcoholic Fatty Liver Disease and Cardiovascular Risk in Children. Children (Basel). 2017 Jul 7;4(7):57.

25. Ahmad MU, Khalid N, Khan S, Asghar U. Risk of Structural Heart Diseases in Non-Alcoholic Fatty Liver Disease. Pakistan Journal of Medical & Health Sciences 2018;12:187-189.

26. González-Ruíz K, Correa-Bautista JE, Izquierdo M, García-Hermoso A, Dominguez-Sanchez MA, Bustos-Cruz RH, García-Prieto JC, Martínez-Vizcaíno V, Lobelo F, González-Jiménez E, Prieto-Benavides DH, Tordecilla-Sanders A, Schmidt-RioValle J, Perez G, Ramírez-Vélez R. Effects of an exercise program on hepatic metabolism, hepatic fat, and cardiovascular health in overweight/obese adolescents from Bogotá, Colombia (the HEPAFIT study): study protocol for a randomized controlled trial. Trials. 2018 Jun 25;19(1):330.

27. VanWagner LB, Wilcox JE, Ning H, Lewis CE, Carr JJ, Rinella ME, Shah SJ, Lima JAC, Lloyd-Jones DM. Longitudinal Association of Non-Alcoholic Fatty Liver Disease With Changes in Myocardial Structure and Function: The CARDIA Study. J Am Heart Assoc. 2020 Feb 18;9(4):e014279.

28. Golabi P, Sayiner M, Paik J, de Avila L, Mishra A, Younossi ZM. ATHEROSCLEROTIC CARDIOVASCULAR DISEASE SCORE IS AN INDEPENDENT PREDICTOR OF MORTALITY IN PATIENTS WITH NONALCOHOLIC FATTY LIVER DISEASE. Gastroenterology 2019;156:S-1253.

29. Liu Y, Zhong GC, Tan HY, Hao FB, Hu JJ. Nonalcoholic fatty liver disease and mortality from all causes, cardiovascular disease, and cancer: a meta-analysis. Sci Rep. 2019 Jul 31;9(1):11124.

30. Moreno-Del Castillo MC, Sanchez-Rodriguez A, Hernandez-Buen Abad JJ, Aguirre-Valadez J, Ruiz I, Garcia-Buen Abad R, Oliva K, Piccolo J, De Icaza-Del Rio E, Mena-Ramirez JR, Mendizabal-Rodriguez ME, Atkinson-Ginsburg NM, Salazar-Segovia J, Ríos-Zertuche Caceres A, Garcia-Juarez I. Importance of Evaluating Cardiovascular Risk and Hepatic Fibrosis in Patients With Newly Diagnosed Nonalcoholic Fatty Liver Disease. Clin Gastroenterol Hepatol. 2019 Apr;17(5):997-999.

31. Chociej AB, Wasilewska N, Flisiak-Jackiewicz M, Lebensztejn D. Cardiovascular Risk in Children with Nonalcoholic Fatty Liver Disease (NAFLD). Curr Pediatr Rev. 2020;16(4):294-297.

32. Erdol MA, Ertem S, Ertem AG, Demirtas K, Unal S, Karanfil M, Akdi A, Yayla C. Adropin: Connection between Nonalcoholic Fatty Liver Disease and Coronary Artery Disease. Med Princ Pract. 2020;29(1):97.

33. Tawadros A, Bhurwal A, Reja D, Pioppo L, Makar M, Kabaria S, Rustgi VK. NAFLD and ischemic stroke: prevalance and effects on outcomes. Gastroenterology 2020;158:S-1431-S-1432.

34. Pennisi G, Di Marco V, Buscemi C, Mazzola G, Randazzo C, Spatola F, Craxì A, et al. Interplay between non-alcoholic fatty liver disease and cardiovascular risk in an asymptomatic general population. J Gastroenterol Hepatol 2021;36:2389-2396.

35. Xu J, Dai L, Zhang Y, Wang A, Li H, Wang Y, Meng X, et al. Severity of Nonalcoholic Fatty Liver Disease and Risk of Future Ischemic Stroke Events. Stroke 2021;52:103-110.

36. Forlano R, Mullish BH, Nathwani R, Dhar A, Thursz MR, Manousou P. Non-alcoholic fatty liver disease and vascular disease. Current Vascular Pharmacology 2021;19:269-279.

37. Han AL. Association of Cardiovascular Risk Factors and Metabolic Syndrome with non-alcoholic and alcoholic fatty liver disease: a retrospective analysis. BMC Endocr Disord. 2021 May 1;21(1):91.

38. Koulaouzidis G, Charisopoulou D, Kukla M, Marlicz W, Rydzewska G, Koulaouzidis A, Skonieczna-Żydecka K. Association of non-alcoholic fatty liver disease with coronary artery calcification progression: a systematic review and meta-analysis. Prz Gastroenterol. 2021;16(3):196-206.

39. Maniak AM, Janardhan SV, Aloman C, Reau N. Non-alcoholic fatty liver disease (NAFLD) is an underrecognized comorbidity in patients presenting with myocardial infarction. American Journal of Gastroenterology 2021;116:S517.

40. Jin C, Bi S, Deng M, Sheng J. Non-alcoholic fatty liver disease impcat on cardiovascular disease death: a population-based study. Journal of Hepatology 2022;77:S165.

41. Ishido S, Tamaki N, Takahashi Y, Uchihara N, Suzuki K, Tanaka Y, Miyamoto H, et al. Risk of cardiovascular disease in lean patients with nonalcoholic fatty liver disease. BMC Gastroenterol 2023;23:211.

Table S3 Methodological quality assessment of included studies with NOS					
First author (publication year)	Selection	Comparability	Outcome	Total (0-9)	Quality
Labenz (2020)	☆☆☆☆	☆☆	☆☆☆	9	High
Choi (2022)	☆☆☆	☆☆	☆☆☆	8	Moderate
Chung (2022)	☆☆☆	☆☆	☆☆☆	8	Moderate
Simon (2023)	☆☆☆☆	☆☆	☆☆☆	9	High
Note: NOS, Newcastle–Ottawa Scale.					

Table S4 The other characteristics of included studies				
**First author, year**	**Continents**	**Study subjects**	**Confounders adjustment**	**HR(95%CI)**
Labenz, 2020	Europe	Patients diagnosed with NAFLD/NASH for the first time between January 2000 and December 2015 at 1262 general hospitals in Germany were enrolled	Age, sex, treating physician, type 2 diabetes, arterial hypertension, hyperlipidemia	AF: 2.98 (1.22-7.93)<br>CHD: 3.12 (1.74-5.60)<br>MI: 2.76 (0.80-9.49)<br>Stroke: 0.89 (0.44-1.83)
Choi, 2022	Asia	A total of 5,333,907 individuals aged 20-39 years who underwent health check-ups at the National Health Insurance Company of Korea between January 2009 and December 2012 were included	Age, sex, hypertension, diabetes mellitus, dyslipidemia, heart failure, prior ischemic stroke, prior myocardial infarction, chronic obstructive pulmonary disease, chronic kidney disease, sleep apnea, hyperthyroidism, smoking, alcohol consumption, low income.	AF: 1.47(1.39-1.55)
Chung, 2022	Asia	A total of 6 891 399 adults aged 20 to 39 years who underwent health screening examinations from 2009 to 2012 (index year considered the baseline) from the Korean National Health Insurance System (NHIS) were	age, sex, smoking, alcohol consumption, regular exercise, diabetes, hypertension, dyslipidaemia, chronic kidney disease, and body mass index.	MI: 1.69 (1.61-1.77)<br>Stroke: 1.47 (1.39-1.56)

		included.		
Simon, 2023	Europe	This population- based, matched cohort study used the ESPRESSO (Epidemiology Strengthened by Histopathology Reports in Sweden) cohort between 1966 and 2016 from children and young adults aged ≤25 years	Age at the index date, sex, calendar year, county of residence,education, the number of recorded hospital visits in the 1 year prior to the index date (or corresponding matching date), diabetes, obesity, hypertension, dyslipidaemia, chronic kidney disease and family history of cardiovascular disease before age 50 years), and alcohol use disorder during follow-up (defined as a time- varying covariate)	AF: 3.36 (1.00-11.35)<br>CHD: 3.07 (1.62-5.83)<br>CHF: 3.89 (1.20-12.64)<br>Stroke: 1.43 (0.59-3.43)
Abbreviations: NAFLD, nonalcoholic fatty liver disease; NASH, non-alcoholic steatohepatitis; MI, myocardial infarction; CHD, coronary heart disease; AF, atrial fibrillation; CHF, congestive heart failure.				

Figure S1. **The figure of sensitivity analysis (leave-one-out analysis)**

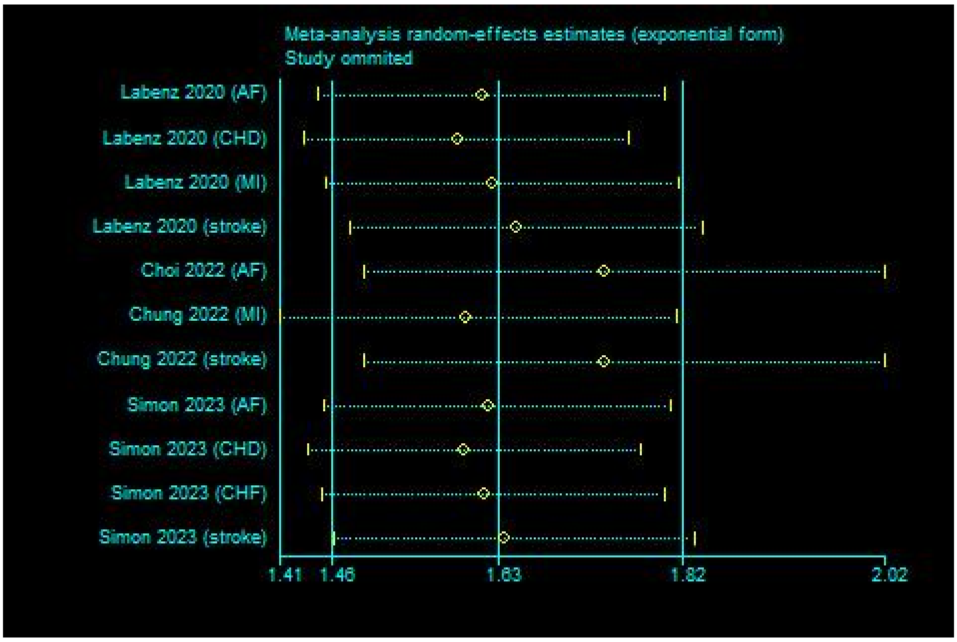

Figure S2. **The Begg's funnel plot for overall results**

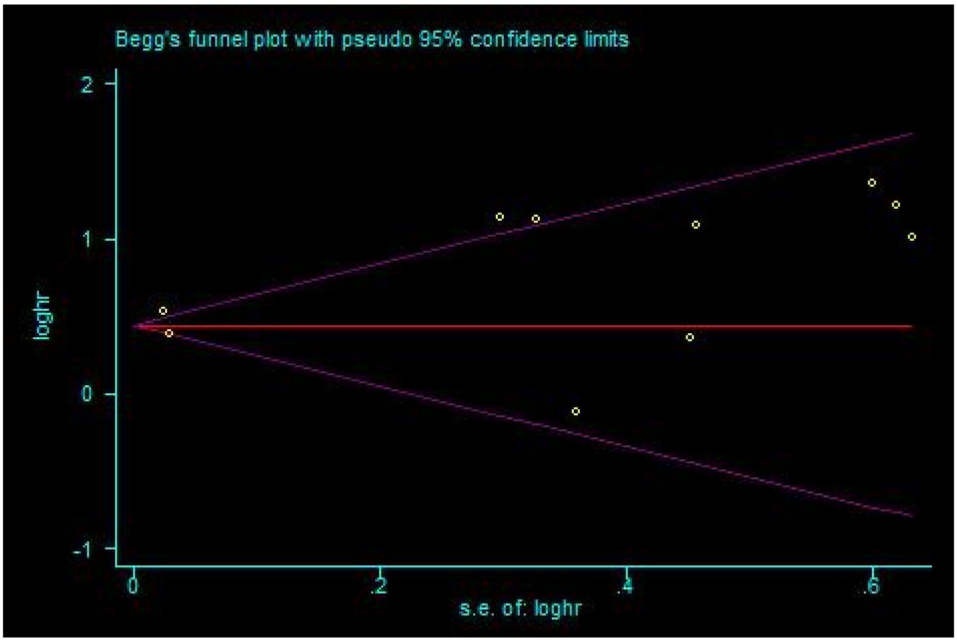

Supplement: Supplementary file 1 [file Datasheet1.pdf]
